# Pollicization of Long Finger After Traumatic Amputation of Thumb and Index Finger

**DOI:** 10.7759/cureus.10760

**Published:** 2020-10-01

**Authors:** Garrett Wegerif, Venus Barlas, Barkat Ali, Gregory Borah

**Affiliations:** 1 Division of Plastic and Reconstructive Surgery, University of New Mexico, Albuquerque, USA; 2 Division of Plastic and Reconstructive Surgery, University of New Mexico School of Medicine, Albuquerque, USA

**Keywords:** pollicization, traumatic amputation, digital amputation

## Abstract

We present a 51-year-old male who sustained a traumatic amputation with a saw of the right thumb, index finger, and carpal bones back to the radius. The amputated digits were mangled and not suitable for replantation. We performed pollicization of the long finger which helped restore a more functional hand. In select situations, this reconstructive option has the advantages of being a single-stage procedure with limited donor site morbidity and no need for microsurgical anastomosis.

## Introduction

Pollicization means the transposition of an adjacent finger to reconstruct a missing thumb. This technique involves the transfer of a finger on a neurovascular bundle, with skeletal fixation, and muscular stabilization in a position which offers opposition and flexion. The reconstructed thumb should have normal sensibility, sufficient mobility, bony stability, proper length, and positioning for function. The indications for pollicization, regardless of the etiology (i.e., congenital or traumatic), depends only on the level of traumatic amputation or aplasia and the availability of adjacent functional digits, usually the index finger.

The first description of use of the long finger pollicization comes from the work of Guermonprez in 1887 [1]. Interestingly, the most common technique today involves pollicization of the index finger, the use of which was not reported until 1903 [2]. Sitting at the crossroads of congenital and traumatic thumb reconstruction, the literature is not very clear for the use of long finger pollicization technique in traumatic amputations. Given the current wide variety of options, from toe-to-thumb transfer to pollicization using either an injured index finger or uninjured long finger, the decision-making and surgical planning are extremely important and individualized [3].

This article presents a challenging case of total thumb and index finger loss secondary to trauma, where we were able to use the long finger to restore a functional thumb. This case highlights several critical points in the surgical planning and evaluating possible options influenced by the patient’s desires and limitations, which resulted in the restoration of a functional thumb. We discuss all the salient features of this case that make it unique.

## Case presentation

This is a 51-year-old male who presented acutely after he sustained a mutilating injury to his dominant right hand while using a large tire saw at work. He had a complete avulsion of the thumb and near-complete amputation of the index finger at the metacarpophalangeal joint. The zone of injury extended proximally to the radius, and severe damage was sustained by the trapezium and scaphoid bones. There was significant soft tissue injury of the radial and dorsoradial aspect of the hand, but his third, fourth and fifth digits were not involved. He had intact sensory and motor function to the ulnar three digits. The amputated thumb was placed on ice and transported with the patient to the hospital.

**Figure 1:**
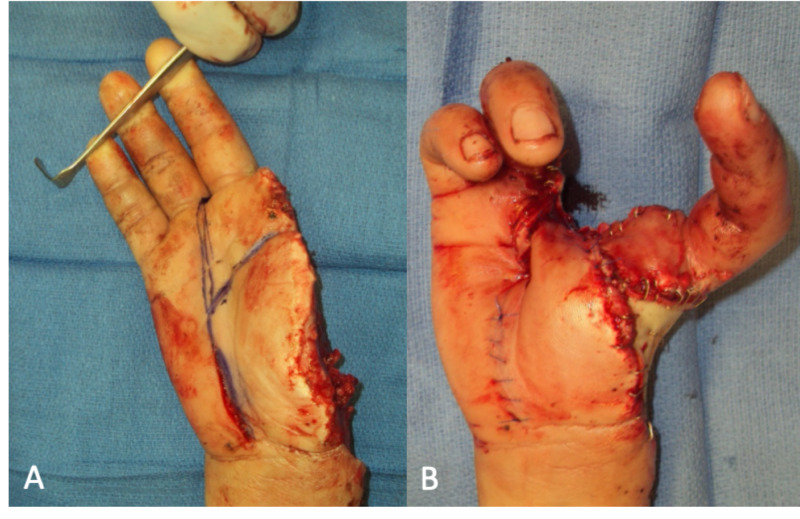
Patient injury at presentation and post-pollicization (A) Amputated thumb and index fingers intraoperatively prior to transposition of middle finger. (B) Result of pollicization of the middle finger.

The patient was taken emergently to the operating room for possible replantation. Careful examination revealed bony amputation of the entire thumb, through the first metacarpal and trapezium. There was multilevel soft tissue injury of the thumb, and hence, it was not suitable for replantation. The second finger had multilevel injuries, with no appreciable blood supply. The index proximal phalanx and the second metacarpal had severely comminuted fractures rendering it nonsalvageable (Figure 1A). A revision amputation of the second finger was performed, and a temporary negative pressure therapy was applied to the damaged radial aspect of the hand.

Multiple reconstructive options were discussed with the patient, including simple soft tissue coverage with a skin graft, osteoplastic reconstruction with a toe-to-thumb transplant, and a single-stage pollicization of the middle finger to reconstruct the thumb. The patient had a significant medical and social history including his active tobacco and cocaine use, in combination with poor glycemic control, which put him at increased risk of wound complications with any surgical intervention. We discussed the utility of a great toe-to-hand transplant for thumb reconstruction, but being a laborer, the patient declined to sacrifice his foot function in addition to his already significant hand disability. The patient wished to proceed with the option that could optimize the function of his hand while requiring the fewest surgical interventions and the quickest return to work.

Investigations

Significant labs on presentation included HbA1c of 11.0 (normal: 4.4-5.6), glucose of 269 (normal: 79.6-114), and albumin of 3.1 (normal: 3.4-4.7).

**Figure 2:**
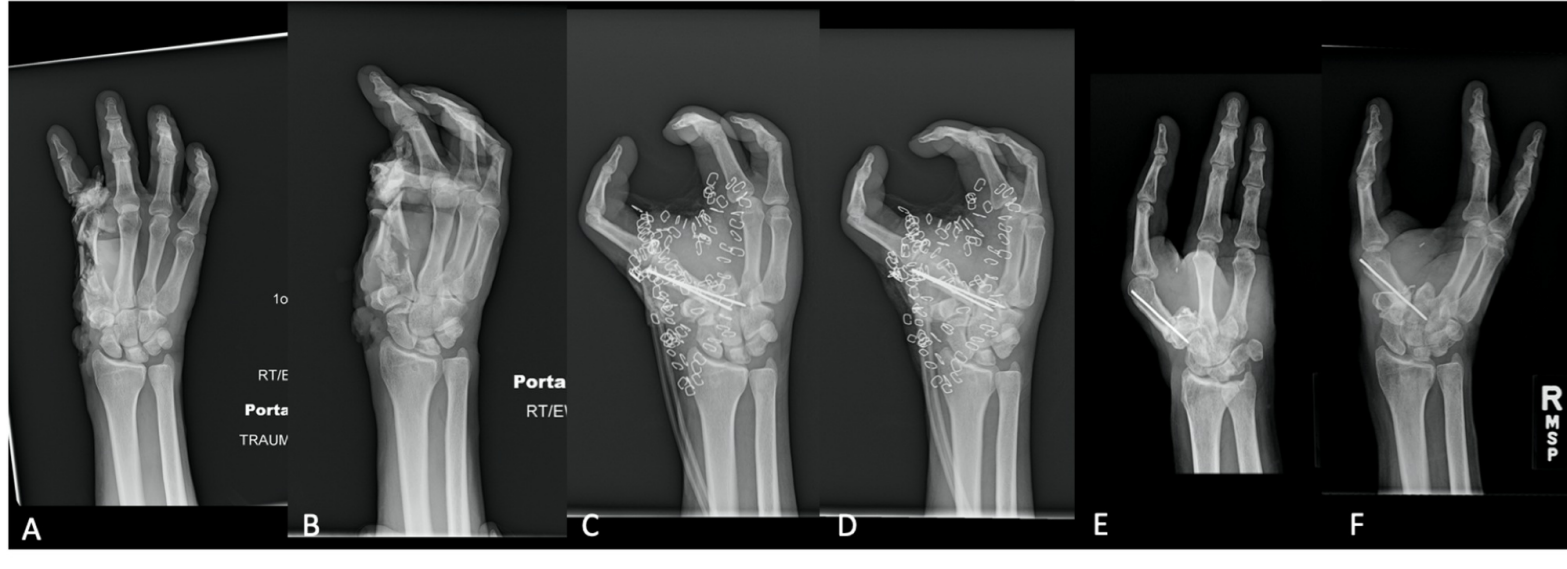
Radiograph images of patient's injury before and after pollicization (A) AP: Injury at presentation. (B) Oblique: Injury at presentation. (C) AP: After pollicization. (D) Oblique: After politicization. (E) AP: six-month follow-up. (F) Oblique: six-month follow-up. AP: anterior-posterior.

Plain radiographs of the right hand demonstrated a complete avulsion of the thumb and severely comminuted fractures of the second metacarpal and second proximal phalanx, as well as injury to the trapezium and scaphoid (Figure 2).

Treatment

Prior to performing pollicization, management of the soft tissue injuries on the hand needed to be addressed. Three days after the initial injury, an additional surgical washout and exploration were undertaken. A ray amputation was performed of the remaining comminuted second metacarpal, and a repair of the extensor tendon laceration of the third finger was done. We injected 80 units of Botox at the base of the remaining digits and throughout the hand to optimize perfusion. The open wound was covered with a dermal matrix layer (Integra Template), and negative pressure wound vacuum-assisted closure (VAC) therapy was begun. The patient was discharged home.

After extended discussions of the patient’s various reconstructive options, nine days later, the patient underwent pollicization of the long finger. Longitudinal skin incisions were placed on the dorsal and palmar surfaces of the hand just ulnar to the third metacarpal, taking care to incorporate the superficial veins of the middle finger. Given the extensive soft tissue injury, a carpal tunnel release was concomitantly performed. Sharp and blunt dissection was used to separate the third finger and intermetacarpal carpal ligaments from the fourth finger and metacarpal, ensuring to include the intact neurovascular bundle with the third finger into the palm. This combined volar/dorsal dissection was carried down to the midportion of the third metacarpal. Capillary refill was continuously checked during this dissection to ensure the third finger remained well perfused.

An angled osteotomy was then performed through the midportion of the third metacarpal, and an attempt was made to transpose the long finger onto the base of the trapezoid. However, there was insufficient length on the pedicle, and the decision was made to transpose the long finger to the capitate instead. An oscillating saw was used to perform an osteotomy on the radial side of the capitate. The third finger was then transposed without tension on the pedicle. The neo-thumb and fourth/fifth fingers could easily be brought in opposition in this position. Two 0.62 K-wires were used to secure the third metacarpal to the capitate and showed excellent bony coaptation.

With the neo-thumb in this transposed position, it was noted that the extensor tendon of the previous third finger was redundant, and since this tendon had been previously repaired at the second operation, the decision was made to excise the redundant portion of the tendon and perform a direct shortening repair. The wound was closed with a combination of local tissue rearrangement and full-thickness skin graft from the lower abdomen (Figure 1B). 100 units of Botox was injected around the radial and ulnar arteries in hopes of preventing vasospasm and maximizing perfusion. A negative pressure wound VAC was applied, and the right hand was placed in a volar one-step splint. Only mild venous congestion was noted at the end of the procedure, and the patient was discharged home the same day.

Outcome and follow-up

**Figure 3:**
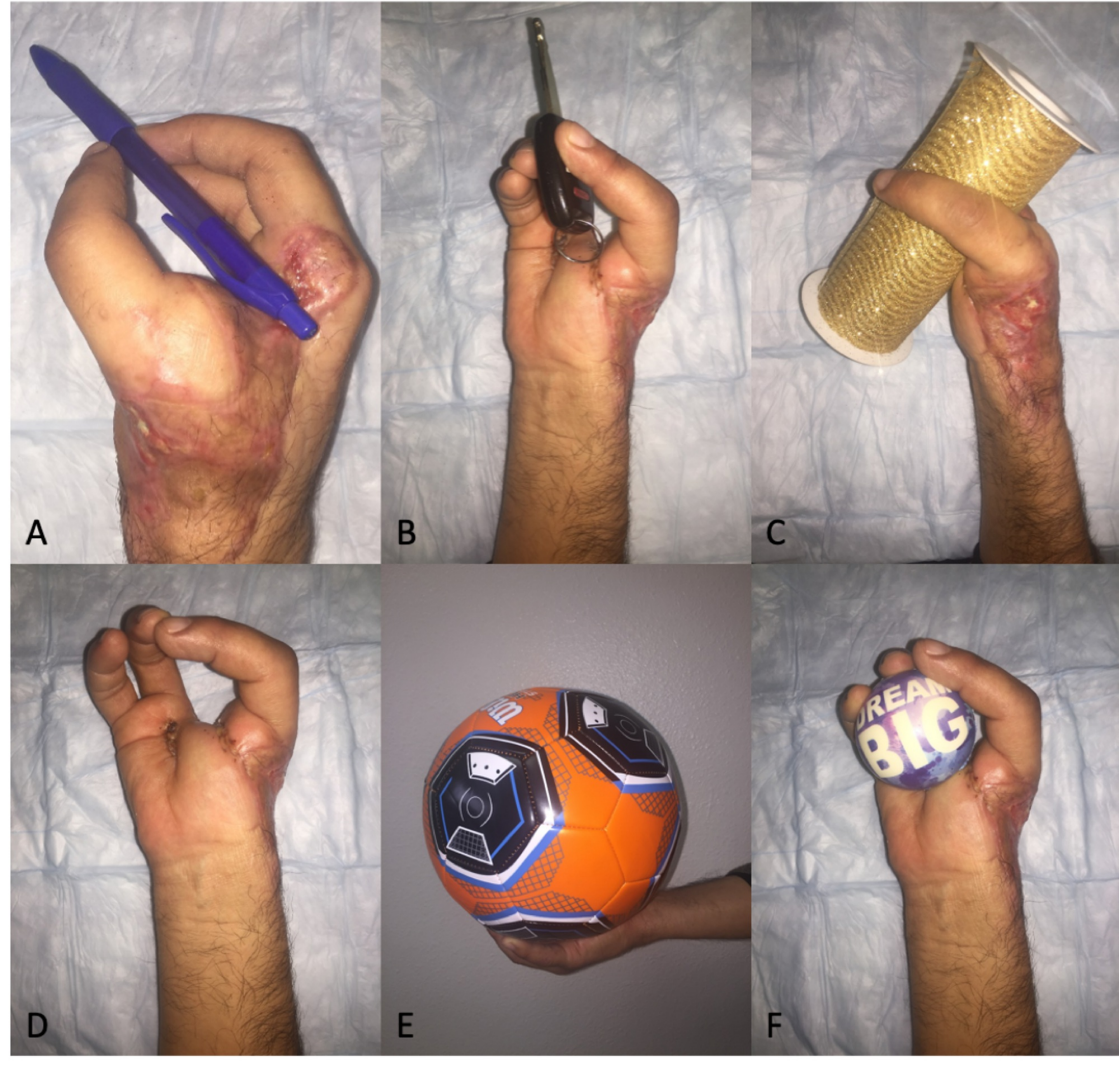
Six-month follow-up gross motor functions (A) Pen hold. (B) Key pinch. (C) Gross grasp. (D)  Firm pinch. (E) Gross hold. (F) Firm hold.

There was no vascular compromise of the pollicized digit, and the patient reported sensibility almost immediately. He began physical therapy at two weeks post-operative. There was some superficial skin necrosis on the hand that required additional skin grafting approximately two months after pollicization. At 6 months follow-up, his wounds had completely healed. His pain level was 0 out of 10 at rest and with most exercises and activities. The right thumb’s metacarpophalangeal joint’s degree of flexion was 50, and the interphalangeal joint’s degree of flexion was 22. His right-hand grip strength was 30 pounds, compared to 74 pounds on the left. Three-point pinch strength was 5 pounds on the right and 18 pounds on the left. Lateral pinch strength was 5 pounds on the right and 19 pounds on the left. His initial QuickDASH (Disabilities of the Arm, Shoulder, and Hand) score was down to 48% from 68% initially at two weeks post-operative. He performed gross grasp, fine grip, key pinch, pen hold, and firm pinch (Figure 3). He was pleased with the overall cosmetic and functional result. There was mild numbness on the radial aspect of the ring finger and dorsal aspect of the base of pollicized thumb, but otherwise sensation was intact to light touch.

## Discussion

Creation of a functional and aesthetically pleasing thumb presents a formidable challenge to a reconstructive surgeon. The default in thumb reconstruction is replantation. If replantation cannot be performed or fails, several other surgical methods can be used to reconstruct the thumb. The optimum technique depends on the level of amputation, age, comorbidities, patient occupation, and patient preference [4].

**Figure 4:**
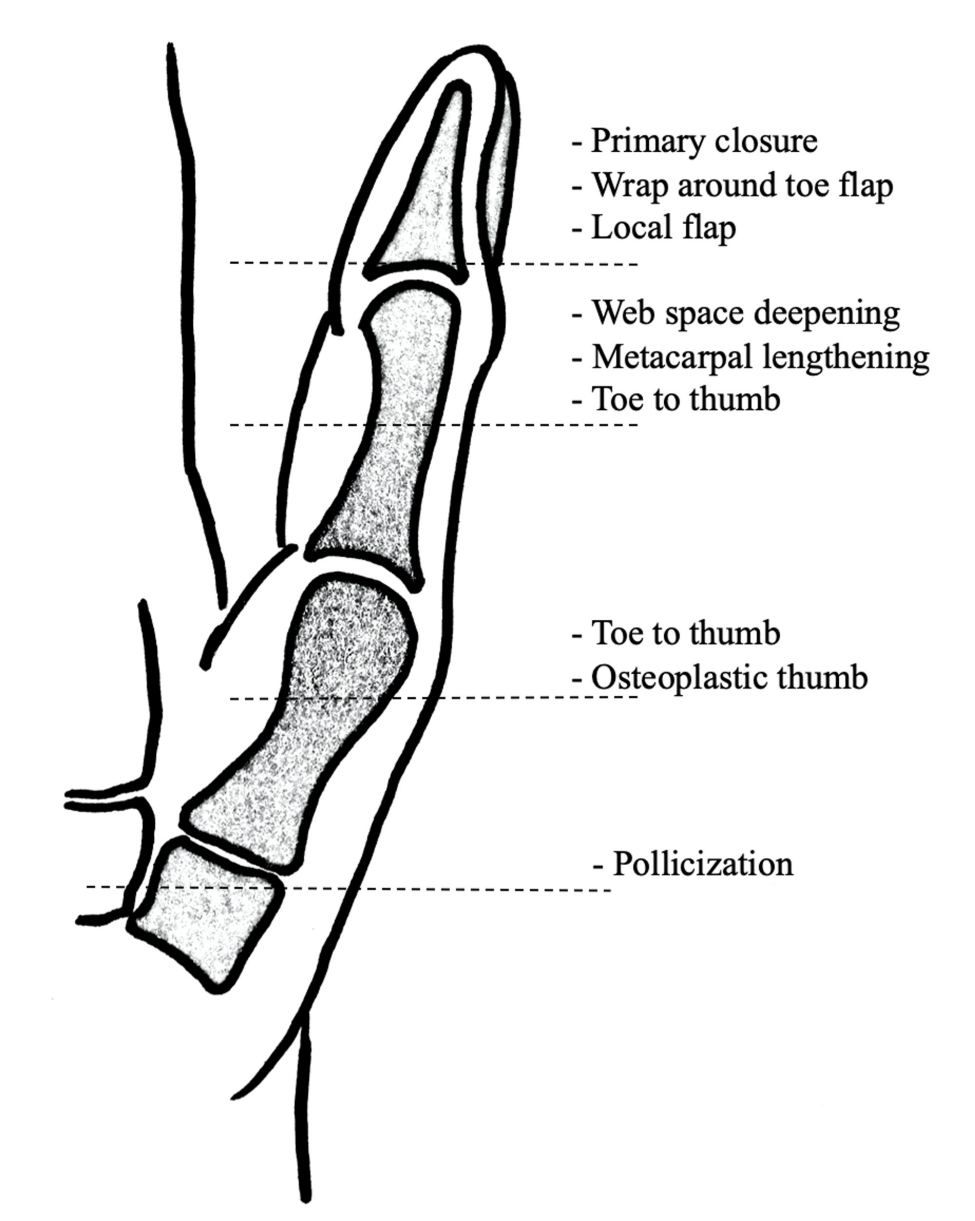
Level of thumb injuries and corresponding surgical option(s) Figure illustrated by author Barkat Ali, MD, University of New Mexico Health Sciences Center.

The classic indication for thumb reconstruction using the pollicization technique is amputation at the carpometacarpal level or proximal, as shown in Figure 4. If the index finger is available, it is the first choice for pollicization [5]. Although, Michon et al. has used intact ring fingers instead of index fingers in post-traumatic thumb amputations [6]. If the index finger is proximally injured and distally viable, the distal intact index finger can be used to reconstruct the thumb [7]. If the index finger is not available, such as in our patient, then use of the long finger should be considered for thumb reconstruction. However, because of the popularity of microsurgical techniques, the indications for pollicization using an intact middle digit have not gained much usage, except when the digit is proximally damaged and distally viable [7]. The acceptable indications for pollicization in trauma are when in addition to thumb amputation, the index finger is injured beyond reconstruction and the remaining stump is of inadequate size to be of any functional benefit or the amputation occurs through the carpometacarpal joint with an intact index finger [8].

If replantation is not an option and amputation is proximal to the metacarpophalangeal joint, then pollicization is the procedure of choice. Pollicization is the safest procedure, shortest to perform, and quickest to obtain results. With the reduced total rehabilitation period, swift restoration of thumb function, easy dissection in the absence of scar tissue, and cost-effectiveness are some of its other inherent advantages [9]. Secondary revision procedures often become necessary in the presence of palmar wounds, severe crush injuries, and massive contamination in the view of risks of infection [7,8]. Toe-to-thumb transfer is a miraculous operation, but it is a lengthy operation and carries the risk of loss of transferred tissues (5%-15%). Because of the required vascular and nerve anastomosis, there is less predictable post-operative sensory and motor recovery. The donor site morbidity of moving a great toe and the attendant physical therapy required is not insignificant. It is for all of the above-stated reasons that our patient chose pollicization.

Some of the technical pearls during the transfer of an intact adjacent digit for thumb reconstruction include preservation of the carpometacarpal joint and thenar muscles and maintenance of the first web space, either with inset of pollicized digit or by means of a flap later. Also, the segment being transferred must have sufficient length to allow opposition with the remaining fingers after the transfer and offer good distal sensibility. At least one digital artery must be intact, and the neighboring finger must retain at least one feeding artery after the transfer [10]. In our case, the amputation was at the carpometacarpal joint with the scaphoid and trapezium bones damaged. The transposed pedicle long finger metacarpal was fused with the capitate using K-wires.

The current literature is not clear about the optimum situation for thumb reconstruction with heterotopic replantation or injured long finger pollicization. Sallis described pollicization using the long finger, but the donor digit was an injured finger [11]. Weinsweing et al. described four cases of pollicization for trauma, where three cases used the long finger [4]. Brunelli and Brunelli described thumb reconstruction in 126 patients with trauma, where pollicization constituted 38 of them, two of which were with an injured long finger [9]. Stern and Lister described 19 pollicizations, 12 of which were abnormal digits (i.e., previous injury to bone or soft tissue or partial amputation) [12]. None of their patients had a completely normal long finger used for pollicization. Dijkstra described the series of 25 thumb reconstructions, of which seven were treated with pollicization. Of note, injured finger remnants were used for pollicization, but only three were done with long finger [13]. Matey and Peart described three pollicizations. Two patients had heterotopic replantations, and one patient had pollicization of a partially injured long finger [14].

We routinely acutely use botulinum A toxin injections in severe traumatic or crush injuries of the hand. Laarakker and Borah described dramatic improvements in digital survival in hand and finger injuries with early Botox injections into the affected fingers and adjacent to radial and ulnar arteries [15]. Botox use for vasospastic disease of the hand is well-described by Neumeister in multiple settings [16]. Despite the mangling nature of our patient’s injury, vascular compromise was not seen. In addition, Botox injections are noted to reduce pain in the hand from injuries, which makes aggressive occupational therapy more effective.

Although there are not direct reports of functional outcomes following pollicization for trauma, functional outcomes after post-traumatic thumb reconstruction in comparison with non-reconstructed thumb have been studied. The findings favor improvement in hand function after thumb reconstruction relative to amputated non-reconstructed thumbs [17,18]. Similarly, our patient demonstrated all essential hand functions.

## Conclusions

Creation of a functional and aesthetically pleasing thumb in patients with hand trauma is a formidable challenge. Pollicization is the reconstructive technique of choice for thumb amputations at or proximal to the carpometacarpal joint. Adjacent finger pollicization is a safe and fast procedure that gives early functional results when done properly. However, if the index finger is also injured and unsalvageable, then one should not hesitate to use the long finger for pollicization.
